# Supplementary material for: Genome-Wide Association Studies Reveal That the Abietane Diterpene Isopimaric Acid Promotes Rice Growth through Inhibition of Defense Pathways
Source: Int J Mol Sci. 2024 Aug 23;25(17):9161. doi: 10.3390/ijms25179161 (PMC11395554; doi:10.3390/ijms25179161)
Supplement: Supplementary file 1 [file ijms-25-09161-s001.zip › ijms-3153042-supplementary.pdf]

International Journal of *Molecular Sciences*

Article

Genome-Wide Association Studies Reveal That the Abietane Diterpene Isopimaric Acid Promotes Rice Growth Through Inhibition of Defense Pathways

Xiaomeng Luo †, Liping Bai †, Jiaqi Huang, Luying Peng, Juan Hua * and Shihong Luo *

College of Bioscience and Biotechnology, Shenyang Agricultural University, Shenyang 110866, China
* Correspondence: huajuan@syau.edu.cn (J.H.); luoshihong@syau.edu.cn (S.L.)
† These authors contributed equally to this work.

Citation: Luo, X.; Bai, L.; Huang, J.; Peng, L.; Hua, J.; Luo, S. Genome-Wide Association Studies Reveal That the Abietane Diterpene Isopimaric Acid Promotes Rice Growth Through Inhibition of Defense Pathways. *Int. J. Mol. Sci.* **2024**, *23*, x. https://doi.org/10.3390/ijms25179161

Academic Editor: Jong Seong Jeon

Received: 27 July 2024
Revised: 18 August 2024
Accepted: 19 August 2024
Published: 23 August 2024

Copyright: © 2024 by the authors. Submitted for possible open access publication under the terms and conditions of the Creative Commons Attribution (CC BY) license (https://creativecommons.org/licenses/by/4.0/).

Table S1. Formula of rice Hoagland nutrient solution.

**Chemical component**	Standard solution g/L
**$Ca(NO_3)_2 \cdot 4H_2O$**	23.8512
$NH_4H_2PO_4$	1.4952
KNO_3	51.561
$MgSO_4$	5.9939
H_3BO_3	0.2993
$MnCl_2 \cdot 4H_2O$	0.201
$ZnSO_4 \cdot 7H_2O$	0.0451
$CuSO_4 \cdot 5H_2O$	0.0262
$Na \cdot EDTA \cdot 2H_2O$	1.6602
$FeSO_4 \cdot 7H_2O$	1.2455

Table S2. The coding sequences of *OsASC1* and *OsBUD13*.

>LOC_Os02g37080.1（OsASC1）
ATGGGGGTCGCGGCGGCGGCGGGCCGGCTCCTGGCGGCGGTGGAC
TGGGAGCGGGAGGCCTACCCGGCGTACCGCGACTTCTTCGCGCTCC
CCTTGTTCGCCGTCTTCTTCCTCGTCGTCCGCTACCTCCTCGACTGC
TTCGTCTTCGAGTGGATTGGAAGAAAACTTATATTTGGAAAGGAAA
AGGTTGATTATGAGAAGGAGGAAACAAGAAAGAAGATAAGAAAAT
TTAAGGAATCAGCTTGGAAATGTGTCTATTTCCTATCTGGAGAGATT
TTATCTTTGTCAGTTACATATAATGAGCCTTGGTTCACAAACACTAA
ATATTTCTGGGTGGACCTGGTGACCAGGTTTGGCCTGACCAAAAGA
TAAAATGGAAACTTAAGGCTGTCTATATGTATGCTGCTGGATTCTACA
CATATTCCATATTTGCACTTATGTTTTGGGAAACAAGGCGTTCAGATT
TTGGCGTGTCAATGTCACATCATGTTGCAACTGTTGCGCTGATAGTTT
TATCTTATGTGTTCAGGTTTGCTAGAGTTGGCTCAGTAGTATTGGCAAT
TCATGATGCAAGTGATGTGTTCCTGGAAGTAGGGAAAATGGCCAAAT
ATAGCCATTGTGATTTGCTTGCCAATGTTGCATTTCTTCTTTTCGTCGT
TTCATGGGTTCTTCTCCGTCTCACATATTTCCGTTCTGGATTCTCAGA
AGTACAAGCTATGAAGTCTTGTTGACCTTGGACAAGAAGAAGCACAA
TTTTGATGGTCCTATATATTACTATGTGTTCAATTCTCTCCTATTTTCACT
ACTTGTTCTTCACATATATTGGTGGGTTCTGATATATCGGATGCTTGTGA
GACAAATCAAGACAAGAAATGTTGGAGATGATGTTCGATCTGACTCTG
AAGGTGAAGACGAGCATGAAGATTGA
>LOC_Os08g08080.1（OsBUD13）
ATGGCGACGAAGCAGCAGGACGCTTCGACGTCCGGCGCGGCCATGT
CCATGAAGGAGTACCTCAAGAGGTACCAATCCGACGACGCGCAGGG
CAAGGCCAAGAAGAAGAAGAAGAAGAAGCCCAAGCCCGCGGCCGC
CGTGGGCGGAGGTGTGCTTATCGTCGACGAGGACCCCACGTGGCACA
AGCCCGTCCAGATCGAGGAGGAGCCCGCGTCGTCCGGGGATGAGAG
ACCGTTGGTGGACGAGGACATCGAGGTCAAGCGGATGCGCCGCCTGG
AGGCGATTCGCGCGGCGCGGCCGTACAATGCTATCGCTGAGGACGGA
AGCGGGTGGGTCACCGTGGCCGCCCCCGAGGACTCCGATGGTGGTTT
AACCGGTCGCCGCCGCAGAAATGACACGCCCTCACCGGAGCGCGGG
GGTGCTGGAAGGAAGGATCTGTCTCCTCCGCGGCGGAGGCAGCGGC
AGGATACGCCCTCGCCGGACCCGAGAGATGCTGCGGGTAAGGATCTG
TCGCCGCCGAGGCAGAGGCGAAGGCGGCAAGACACGCCGTCACCAA
AGGGTAGTGAGGTAGCTGGGGGGCATGATGATTTGTCACCACCGCGGA
AGTCTAGGCAGCAACGAGACCCCTCTCCTCCAGCAGGCTCTCTCGCCA
TGACTCCAAGGAATCCCAGGACATTTCACCACCACGAAGGCGTACCAG
GCATGACTCAGAGGAGCCACAGGACCTCTCTCCACCACGCCGGAAGG
GGCGGCATGATTCTGAAGAACCCAAAGACCTCTTGCCACCATGGAGGC
GCGTGAGGCATGATTCTGAGGAACCCAAAGACCTCTCGCCACCACGGA
GGCGCACGAGACATGATTCAGAAGAGCCCGAAGACCTCTCTCCCCCAC
GCAGACGGACGCGGCACGACTCTCATGAACCCAAGGACAAATTGCCAC
CACTGAAAAGGCAGGCTTTGGGTGATGGGGACATTTCACCTCCAAGGA
AGGGTAGGAAGGAAGTGGCTCCGAAGGAGGTGAGGAAAGCTGGGTTG
ATGACAGCAGAGGAAGTTAAAGAGGACATCAGGAAGATTAAGGAGGAT
GAGAGGCTCAAGTTTGCTGCACAGGATCCCTCAGTGATTGGGAAAGGG
GCAAAAGCAGTATTCCGAGATAAACAAGGAAAACGAATAAGTGAAGAA
GATATGAGGAAGGCAAAGGAAGACACGAAACCAAAGGAAATACATAT
AGAATGGGGTAAAGGGTTGGTGCAGAAGCGAGAAGCTGAGGCTAGA
TTACAGGAGCTTGAATCTGAAAAGAGTAAGCCATTTGCACGGACAAGGG
ATGATCCTGAGCTCGACACCATGCTAAAAACAGAATCCGGTGGGGTGAT
CCTATGGCTCATCTTGTCAAGAGGAATGATACAGACCTCCTTCTGGAGG
ACTTGGGAGATGATGAAAAGATGAAAGAATCTGGCTTCATTGTTCCCCA
AAATATACCTAGTCACAGCTGGCTGAAGCGTGGTGTAGATCCTCCTCCAA
ACCGCTACGGCATAAAGCCTGGTCGTCATTGGGACGGAGTGGATCGCA
GCAATGGATTTGAGAAGGATATGTTTAAGCTGAAGAACGAGAAGCAAGC
AACGGAGCAAGAGGCCTACCTTTGGTCTGTCGCAGATATGTGA

Table S3. The primers used in this experiment.

Primer name	Sequence (5'-3')
OsASC1-GFP-F	ttgatacatatgcccgtcgacATGGGGGTCGCGGCGGCG
OsASC1-GFP-R	gctcaccatggatccggtaccATCTTCATGCTCGTCTTCACCTTC
OsBUD13-GFP-F	ttgatacatatgcccgtcgacATGGCGACGAAGCAGCAG
OsBUD13-GFP-R	gctcaccatggatccggtaccCATATCTGCGACAGACCAAAGGT
qOsASC1-F	GCGTTCAGATTTTGGCGTGT
qOsASC1-R	GGCAAGCAAATCACAATGGC
qOsBUD13-F	GGCTGAAGCGTGGTGTAGAT
qOsBUD13-R	CCGTTGCTTGCTTCTCGTTC
actin-7-F	TCACTGCCCTCGCACCAAG
actin-7-R	GCTGGACCCGACTCATCATACTC

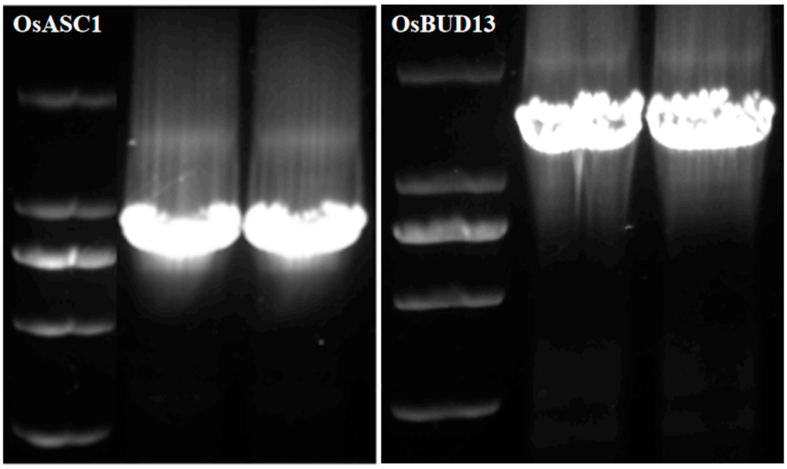

Figure S1. The electrophoresis results of the PCR amplification of *OsASC1* and *OsBUD13*. .

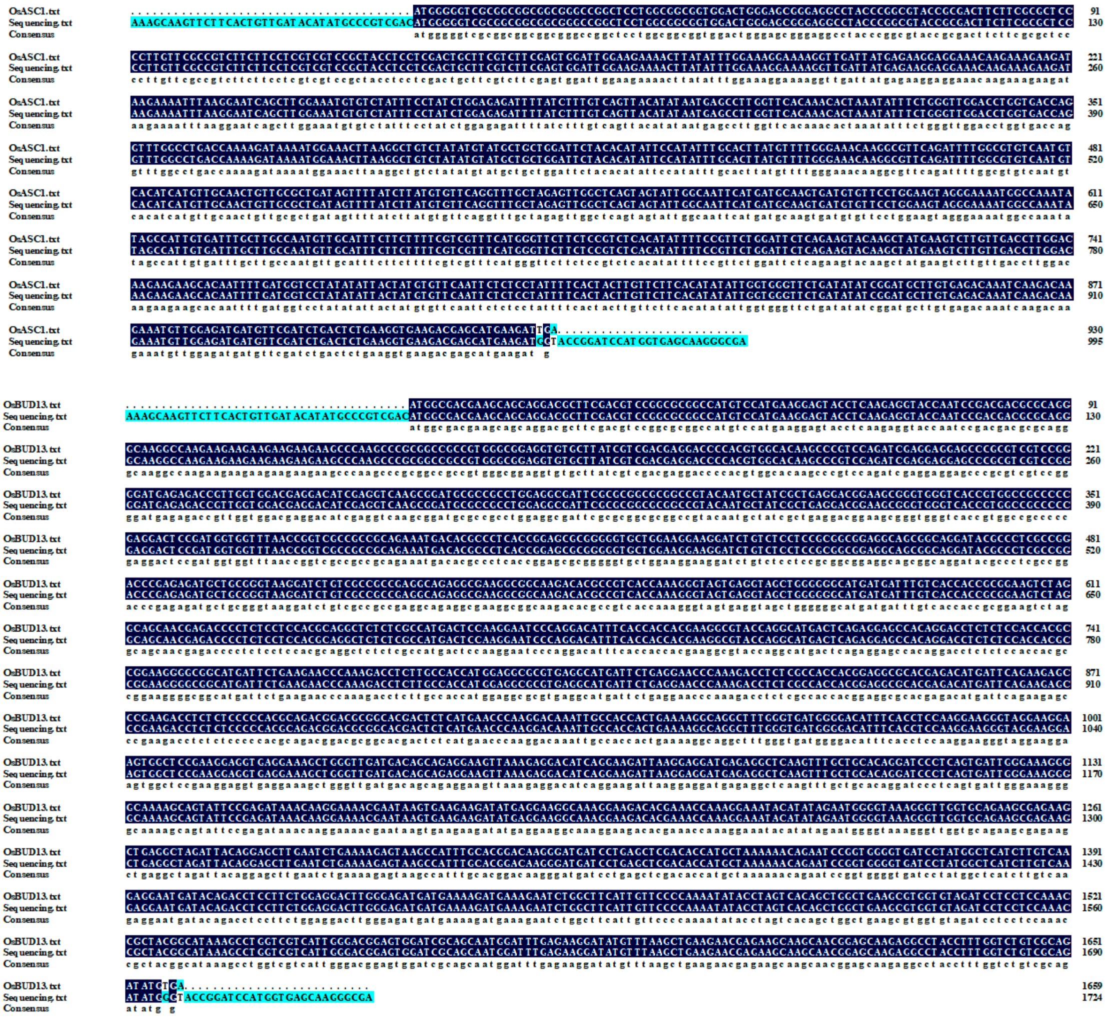

Figure S2. Sequence alignment of *OsASC1* and *OsBUD13*.
